# LncRNA NEAT1 Targets Fibroblast-Like Synoviocytes in Rheumatoid Arthritis via the miR-410-3p/YY1 Axis

**DOI:** 10.3389/fimmu.2020.01975

**Published:** 2020-08-28

**Authors:** Yuejiao Wang, Linxin Hou, Xiaowei Yuan, Neili Xu, Shuai Zhao, Lili Yang, Ning Zhang

**Affiliations:** ^1^Department of Rheumatology and Immunology at Shengjing Hospital of China Medical University, Shenyang, China; ^2^Department of Orthopedics at Shengjing Hospital of China Medical University, Shenyang, China

**Keywords:** rheumatoid arthritis, fibroblast-like synoviocytes (FLSs), NEAT1, miR-410-3p, YY1

## Abstract

LncRNA NEAT1 functions as an oncogene in multiple human cancers. However, its expression and role in fibroblast-like synoviocytes (FLSs) from patients with rheumatoid arthritis (RA) remain unclear. Thus, we investigated the expression of NEAT1 in synovial tissues and FLSs in RA, to determine its role in the development of RA. Quantitative real-time polymerase chain reaction was used to measure the expression of NEAT1. FLS proliferation was evaluated using cell proliferation assays. Flow cytometry was used to analyze cell cycle progression and apoptosis in FLSs. Binding between NEAT1 and miR-410-3p was demonstrated by dual-luciferase assays. We found that NEAT1 was upregulated in synovial tissues and FLSs in RA. Upregulation of NEAT1 promoted cell proliferation, induced S-to G2/M phase transition, and suppressed apoptosis in RA FLSs. NEAT1 directly bound to and negatively modulated miR-410-3p expression, while positively regulating YinYang 1(YY1; a miR-410-3p target). Inhibiting miR-410-3p and upregulating YY1 partially restored the inhibitory role in cell viability induced by the depletion of NEAT1 in RA FLSs. Besides pro-proliferative and anti-apoptotic roles, upregulation of NEAT1 promoted migration, invasion, and inflammatory cytokines secretion in RA FLSs. Taken together, our results suggest that the NEAT1 may serve as a novel diagnostic and therapeutic target for patients with RA.

## Introduction

Rheumatoid arthritis (RA) is a commonly occurring chronic autoimmune disease, characterized by inflammation of the joints, subsequent destruction of cartilage, and bone erosion (1). RA affects ~0.5–1.0% of the global population (2018). Outcomes of patients with RA can be improved by early diagnosis, new drugs and therapeutic regimens. Current therapy against RA includes conventional and biological disease-modifying antirheumatic drugs. Individual or combinations of these drugs currently in use have resulted in the complete or partial clinical remission of majority of the patients suffering from RA (2). However, a subset of patients fail to respond to treatment and exhibit the slow, but persistent, radiographic progression of RA in affected joints (3). Therefore, it is imperative to identify new therapeutic targets for RA.

Fibroblast-like synoviocytes (FLSs) are integral components of the synovial intima. The human synovium comprises 2–3 layers of cells (4). During RA, hyperactivation of FLSs causes the thin synovium to become hyperplastic and form a pannus-like structure (5). The early phase of RA includes the presence of activated FLSs in the synovium accompanied by an aggressive phenotype, such as excessive proliferation, inhibited apoptosis, secretion of proinflammatory cytokines, migration, and invasion (6). These tumor-like characteristics induced by FLSs help distinguish RA from other chronic inflammatory diseases, such as osteoarthritis. However, the mechanisms employed by FLSs in the development of RA remain to be elucidated. Moreover, the current therapeutic strategies do not target activated FLSs. Therefore, understanding the underlying mechanisms in play in abnormally activated FLSs will help to identify novel diagnostic markers and therapeutic targets.

Long non-coding RNAs (lncRNAs) are transcripts longer than 200 nucleotides that are involved in the development of RA by regulating cell proliferation (7), apoptosis (8), migration, and invasion (9) of FLSs. MicroRNAs (miRNAs) constitute another class of non-coding RNAs that are dysregulated in autoimmune diseases (10). LncRNA-miRNA-mRNA interactions are pivotal in various biological phenomena in oncology and immunology wherein lncRNAs have been shown to function as miRNA sponges (11, 12). Recent studies have demonstrated that the lncRNA NEAT1 (nuclear enriched abundant transcript 1) is an oncogene that is overexpressed in multiple malignancies, including colorectal cancer (13), breast cancer (14), and leukemia (15). Shui et al. reported the upregulation of NEAT1 in peripheral blood mononuclear and Th17 cells in patients with RA and during the CD4+ T cells differentiation (16). However, the role of NEAT1 in FLSs in patients with RA is unknown.

We have previously shown that miR-410-3p targets Yin Yang1 (YY1) to regulate cell proliferation, apoptosis, and cell cycle progression in FLSs during RA (17). Thus, we hypothesized that NEAT1 affects cell viability in RA FLSs by targeting the miR-410-3p/YY1 axis.

## Materials and Methods

### Patient Samples

To determine the relative expression of NEAT1, synovial tissues of patients with RA and normal synovial tissues of patients receiving emergent traumatic amputation as healthy controls were collected from the Department of Orthopedics at the Shengjing Hospital of China Medical University. RA was diagnosed based on the 2010 ACR/EULAR criteria for classification of RA (18). All procedures used in this study were approved by the Ethics Committee of Shengjing Hospital of China Medical University.

### Cell Lines and Transfection

Normal and RA affected human FLSs (HFLS and HFLS-RA, respectively) were obtained from Jennio Biotech Co., Ltd. (Guangzhou, China). The HFLS and the HFLS-RA cell lines were cultured in minimum essential medium and Dulbecco's modified Eagle medium (Corning, USA) with high glucose, respectively, 10% fetal bonine serum (Gibco, USA), and 1% penicillin-streptomycin (HyClone, USA). TNF-α (10 ng/mL) (Sigma-Aldrich, St.Louis, USA) was used to stimulate HFLS-RA cells. HFLS-RA cells were transfected with pcDNA-NEAT1, small interfering RNAs (siRNAs), miR-410-3p mimics, inhibitor, or pcDNA-YY1 (synthesized by GenePharma, Shanghai, China) using Lipofectamine 3000 (Invitrogen, USA) as previously described (17). The aforementioned sequences were as follow: has-miR-410-3p mimcis: 5′-AAUAUAACACAGAUGGCCUGUAGGCCAUCUGUGUUAUAUUUU-3′; has-miR-410-3p inhibitor: 5′-ACAGGCCAUCUGUGUUAUAUU-3′; mimics NC: 5′-UUCUCCGAACGUGUCACGUTT-3′ (sense), 5′-ACGUGACACGUUCGGAGAATT-3′ (antisense); inhibitor NC: 5′-CAGUACUUUUGUGUAGUACAA-3′; siRNA#1-NEAT1-Homo-1527 (Cat.No.A10001): 5′-GAUGCUGCAUCUUCUAAAUTTAUUUAGAAGAUGCAGCAUCTT-3′; siRNA#2- NEAT1-Homo-2240:5′-GCAGGUUGAAGGGAAUUCUTTAGAAUUCCCUUCAACCUGCTT-3′;siRNA#3-NEAT1-Homo-452:5′-GGGCUAAUCUUCAACUUGUTTACAAGUUGAAGAUUAGCCCTT-3′.

### Quantitative Real-Time Polymerase Chain Reaction (qRT-PCR)

Total RNA from synovial tissues and cells were extracted with RNAiso Plus reagent (Takara, Japan), and 1 μg was reverse-transcribed to cDNA with a Mir-X™ miRNA First-Strand Synthesis and SYBR® qRT-PCR kit (Takara) and the PrimeScript™ reagent Kit (Takara) for miR-410-3p, NEAT1, and YY1, respectively, in accordance with the manufacturer's instructions. Quantification was performed using an SYBR® Premix Ex Taq™ II kit with the 2^−ΔΔCT^ method, normalized to GAPDH or U6. The PCR primers were synthesized by Sangon Biotech (Shanghai, China) as follows: 5′- GTAATTTTCGCTCGGCCTGG-3′ (forward) and 5′- CACATTCACTCCCCACCCTC-3′ (reverse) for NEAT1; 5′-CGCGAATATAACACAGATGGCCTGT-3′ (forward) for has-miR-410-3p; 5′-AGCCCTTTCAGTGCACGTT-3′ (forward) and 5′-TCTCCGGTATGGATTCGCAC-3′ (reverse) for YY1; 5′-AAGCCTGCCGGTGACTAAC-3′ (forward) and 5′-GCATCACCCGGAGGAGAAAT-3′ (reverse) for GAPDH; 5′-TGGAACGCTTCACGAATTTGCG-3′ (forward) and 5′-GGAACGATACAGAGAAGATTAGC-3′ (reverse) for U6.

### Cell Proliferation

Cell proliferation was measured using the Cell Counting Kit-8 (CCK-8; Promega, USA). Briefly, HFLS-RA cells were seeded in five 96-well-plates with a density of 5 × 10^3^ cells/well. After transfection and incubation for 24, 48, 72, and 96 h, 100 μl of medium and 10 μl of the CCK-8 reagent were added into each well and incubated for 4 h at 37°C. OD_490_ for each sample was measured using a microplate reader.

HFLS-RA cells were seeded into 96-well-plates at a density of 5 × 10^3^ cells/well. After transfection for 30 h, old medium was replaced with 100 μl of fresh medium containing 50 μM EdU (Ribobio, China) and incubated for 18 h at 37°C. The cells were fixed with 4% formaldehyde and treated with Triton X-100 and DAPI for 5 min followed by visualization by fluorescence microscopy. Ratios of EdU-positive cells were calculated for at least random five fields with Image J software.

### Apoptosis and Cell Cycle

HFLS-RA cells were transfected and harvested after 48 h, and processed as per the PE Annexin V Apoptosis Detection Kit (BD, USA). Briefly, 5 μl of the PE reagent and 5 μl of 7-AAD reagent were added into the cell suspensions and incubated for 15 min at room temperature. Finally, we analyzed the cells in different phases using the FACSAria flow cytometer (BD).

HFLS-RA cells were harvested 48 h after transfection and fixed with 75% ethanol at −20°C for at least 2 h. Subsequently, cells were resuspended in the PI reagent (BD). Finally, we analyzed the cells in different phases using the FACSAria flow cytometer (BD).

### Migration and Invasion Assays

HFLS-RA cells were transfected with pcDNA-NEAT1 or siRNAs against NETA1, digested with trypsin, and seeded in high glucose DMEM without FBS in the upper chamber of a 24-well transwell insert or Matrigel transwell insert, respectively. The lower chamber was filled with high glucose DMEM with 10% FBS. After 24 h, HFLS-RA cells that had passed through the membrane were fixed with methanol and stained with crystal violet solution. The cell number per field were calculated with Image J software and the mean values of random five fields were analyzed.

### Enzyme-Linked Immunosorbent Assay (ELISA)

HFLS-RA cells were seeded in a 24-well-plate and transfected with pcDNA-NEAT1 or siRNAs against NETA1 for 48 h. The concentration of TNF-α and MMP-9 in the cell culture supernatants were determined by a sandwich ELISA kit (R&D Systems, Minneapolis, MN) according to the manufacturer's instructions.

### Luciferase Assay

The wild-type and mutant sequences of NEAT1 mRNA containing the putative miR-410-3p-binding sites were synthesized and cloned into the pmirGLO vector (Promega). We then co-transfected HFLS-RA with miR-410-3p mimics or NC and the wide-type or mutant constructs. After incubating for 48 h, the HFLS-RA cells were harvested and subjected to the Dual-luciferase Reporter assay system (Promega) to determine binding between NEAT1 and miR-410-3p.

### Western Blotting

Synovial tissues and FLSs were lysed using RIPA lysis buffer (Beyotime, China). Total protein concentrations were measured using a BCA kit (Beyotime). Western blotting and ECL detection were performed according to standard procedures (17). Primary antibodies against YY1 (1:500 dilution) and GAPDH (1:5,000 dilution) were procured from Proteintech (China). Protein band intensities were normalized using those of GAPDH and the data were analyzed by Image J.

### Statistical Analysis

All the experiments were independently performed in triplicates. Student's *t*-test and one-way analysis of variance were used for comparisons between two groups or multiple groups, respectively. Two-way analysis of variance was used to compare cell proliferation data. GraphPad Prism software (v.7.0, San Diego, CA) was used to analyze. *P* < 0.05 was considered statistically significant.

## Results

### Upregulated NEAT1 in RA

**Figure 1:**
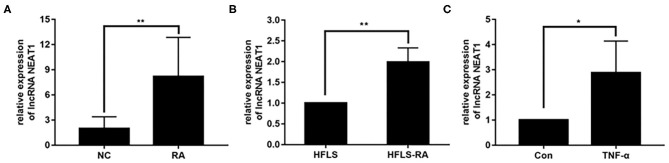
Upregulated NEAT1 in RA. **(A)** The relative expression of NEAT1 was detected in the synovial tissues from patients with RA (*n* = 9) and healthy controls (*n* = 8). **(B)** The relative expression of NEAT1 in HFLS and HFLS-RA cells. **(C)** The relative expression of NEAT1 in HFLS-RA cells with or without TNF-α (10 ng/mL) treatment. All the experiments were independently performed in triplicates. *p < 0.05, **p < 0.01, compared with the relative negative control (NC) groups.

To investigate the potential roles of NEAT1 in RA, we determined the expression of NEAT1 in synovial tissues and cells. qRT-PCR showed that NEAT1 was upregulated in patients with RA, compared to that in healthy controls (*p* = 0.0026, Figure 1A). As compared to the levels in HFLS cells, NEAT1 was overexpressed in HFLS-RA (*p* = 0.0011, Figure 1B). Furthermore, NEAT1 was upregulated in HFLS-RA cells with TNF-α treatment compared to that without TNF-α treatment (*p* < 0.05, Figure 1C), indicating the involvement of NEAT1 in the development of RA.

### Effects of NEAT1 on Cell Viability of HFLS-RA Cells

**Figure 2:**
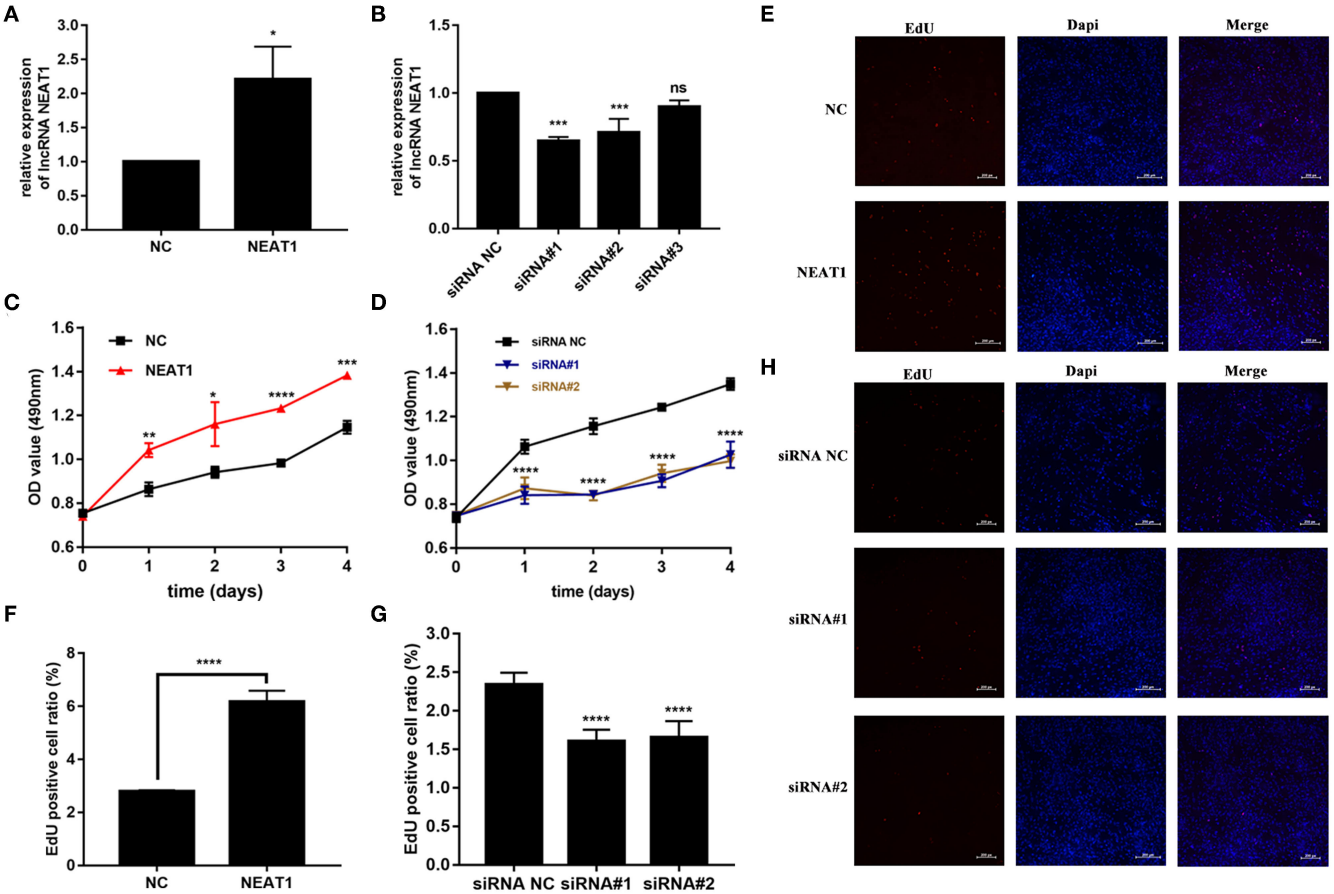
Effect of NEAT1 on the proliferation of HFLS-RA. **(A,B)** The relative expression of NEAT1 in HFLS-RA cells transfected with pcDNA-NEAT1 or siRNAs against NEAT1. **(C,D)** Proliferation of HFLS-RA cells transfected with pcDNA-NEAT1 or siRNAs against NEAT1 for 0, 24, 48, 72, and 96 h. **(E–H)** EdU staining intensities showing the proliferation of HFLS-RA cells transfected with pcDNA-NEAT1 or siRNAs for 48 h. The percentages of EdU-positive cells were calculated using random five fluorescence microscopy fields. All the experiments were independently performed in triplicates. *p < 0.05, **p < 0.01, ***p < 0.001, and ****p < 0.0001, compared with the NC groups.

We performed gain- and loss-of-function assays to manipulate the expression of NEAT1 in HFLS-RA cells. As shown in Figures 2A,B, NEAT1 was overexpressed using the pcDNA-NEAT1 construct in HFLS-RA cells (*p* = 0.0116); we used three siRNAs against NEAT1 to downregulate NEAT1 (*p* = 0.0002, *p* = 0.0006, and *p* = 0.09, respectively), and siRNA#1 and siRNA #2 were selected for further analysis.

**Figure 3:**
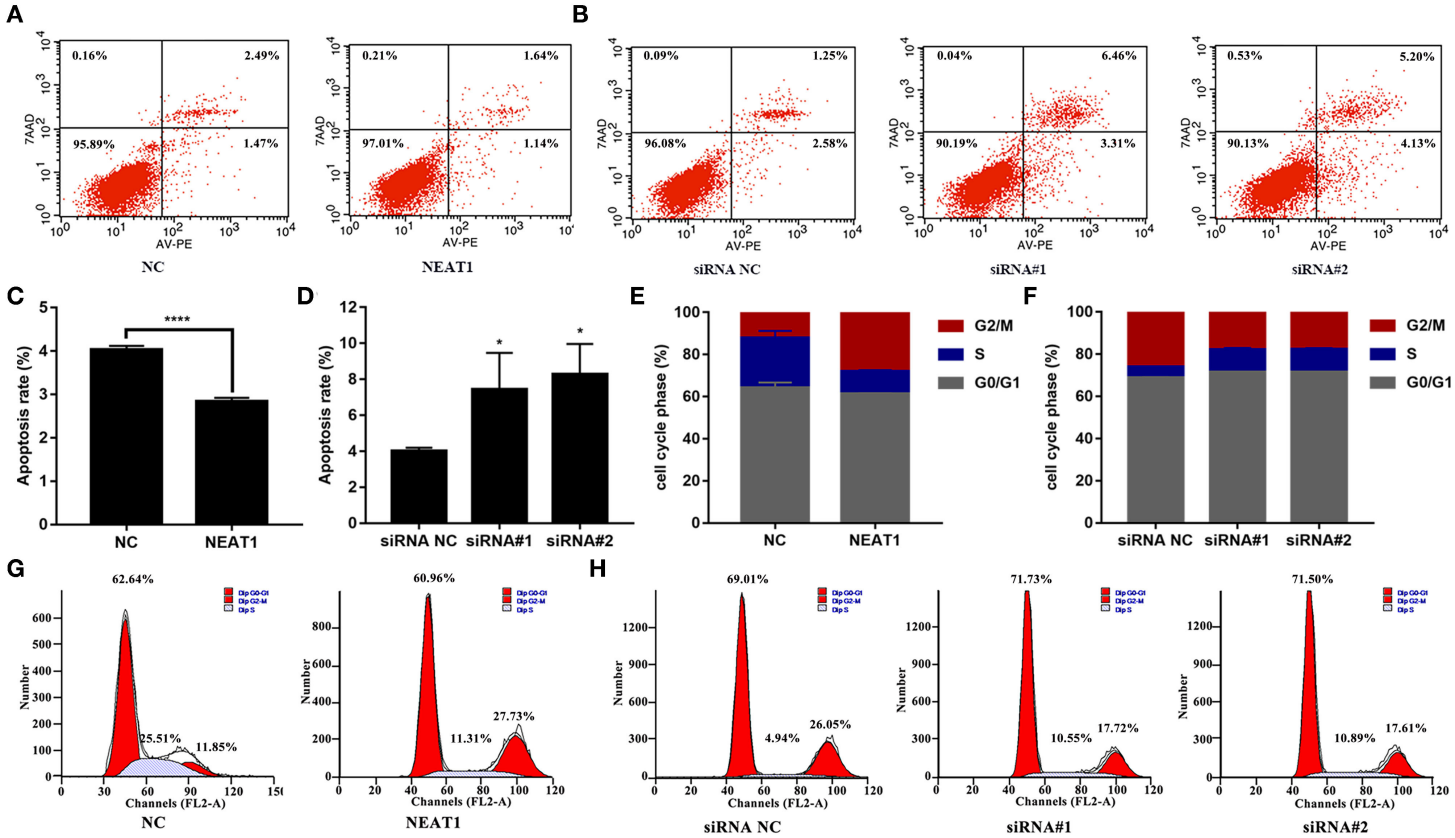
Effect of NEAT1 on the apoptosis and cell cycle of HFLS-RA **(A–D)** Flow cytometric analysis for the rates of apoptosis (%) in HFLS-RA cells transfected with pcDNA-NEAT1 or siRNAs against NEAT1 for 48 h. The percentage of total apoptotic cells were calculated and compared among groups. **(E–H)** Flow cytometric analysis of the percentage (%) of HFLS-RA cells transfected with pcDNA-NEAT1 or siRNAs against NEAT1 for 48 h in different phases. All the experiments were independently performed in triplicates. *p < 0.05, and ****p < 0.0001, compared with the relative NC groups.

The CCK-8 assay showed that NEAT1 overexpression enhanced the proliferation of HFLS-RA cells (*p* < 0.05, Figure 2C), while the downregulation of NEAT1 suppressed the proliferation of HFLS-RA cells (*p* < 0.05, Figure 2D). In accordance with the proliferation assay, EdU fluorescence intensity revealed that DNA synthesis was significantly enhanced in pcDNA-NEAT1-transfected HFLS-RA cells (*p* < 0.0001, Figures 2E,F), while DNA synthesis was inhibited in HFLS-RA cells transfected with siRNAs against NEAT1 (*p* < 0.0001, Figures 2G,H). Upregulation of NEAT1 reduced the percentage of apoptotic HFLS-RA cells (*p* < 0.0001, Figures 3A,C), while downregulation of NEAT1 increased apoptosis (*p* = 0.0446 and *p* = 0.0334, respectively, Figures 3B,D). Flow cytometry showed an increase in the proportion of G2/M phase cells under NEAT1 overexpression (*p* < 0.0001, Figures 3E,G) that are reversed by downregulating NEAT1 (*p* < 0.0001 and *p* = 0.0031, respectively, Figures 3F,H). Taken together, NEAT1 promotes cell proliferation and S-to-G2/M phase transition while suppressing apoptosis in HFLS-RA cells.

### NEAT1 Competitively Binds miR-410-3p to Regulate YY1 Levels in HFLS-RA Cells

**Figure 4:**
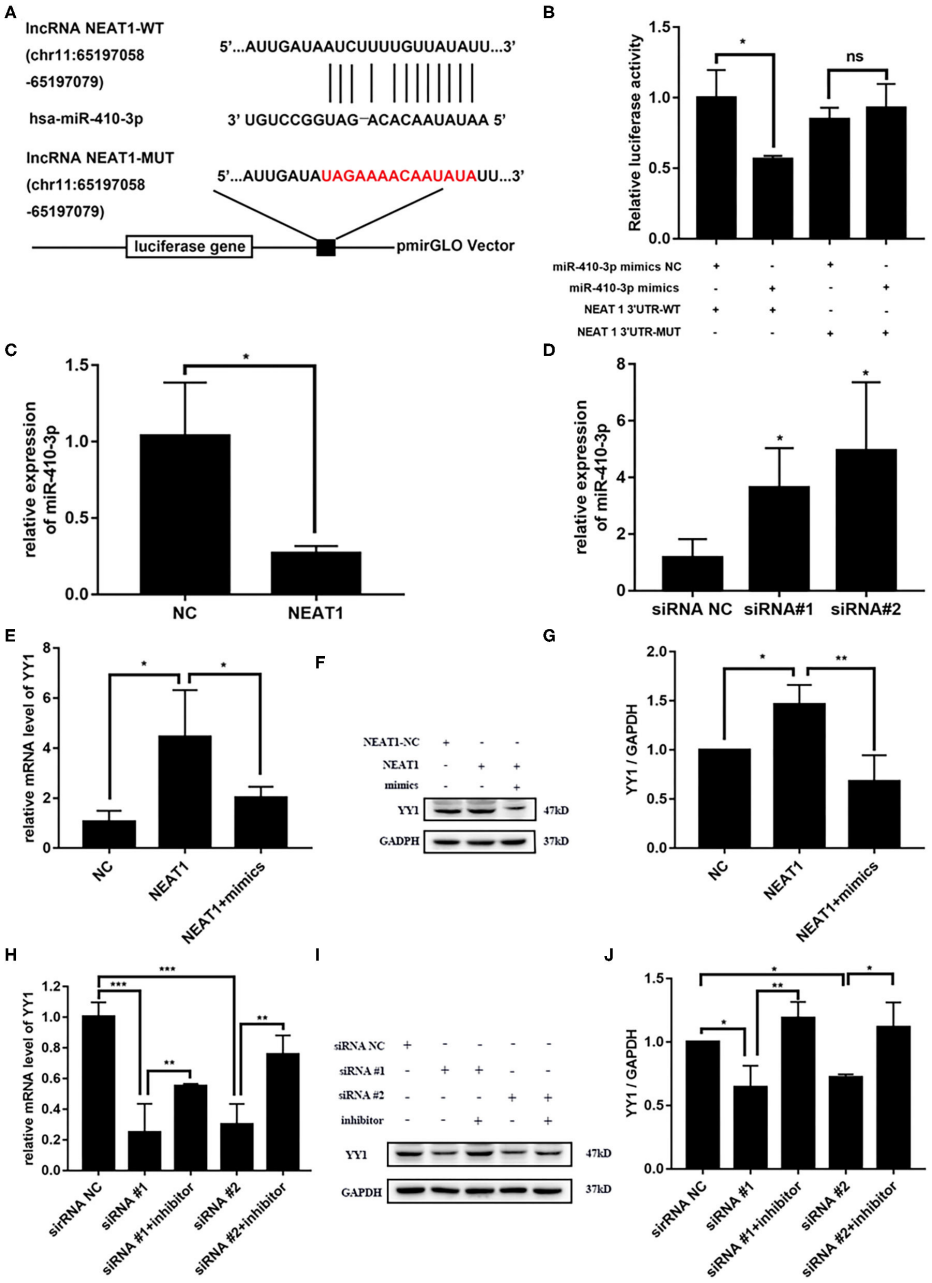
NEAT1 competitively binds miR-410-3p to regulate YY1 levels in HFLS-RA cells **(A)** Binding sites of miR-410-3p in NEAT1 was predicted by ENCORI. **(B)** Interaction between NEAT1 and miR-410-3p was analyzed by dual-luciferase reporter assays. **(C,D)** The relative expression of miR-410-3p in HFLS-RA cells transfected with the pcDNA-NEAT1 or siRNAs against NEAT1. **(E–G)** The relative mRNA and protein levels of YY1 in HFLS-RA cells transfected with the pcDNA-NEAT1 or pcDNA-NEAT1 and miR-410-3p mimics. **(H–J)** The relative mRNA and protein levels of YY1 in HFLS-RA cells transfected with siRNAs against NEAT1 or siRNAs and miR-410-3p inhibitor. All the experiments were independently performed in triplicates. *p < 0.05, **p < 0.01, and ***p < 0.001, compared with the relative NC groups.

NEAT1 functions as a competing endogenous RNA (ceRNA) in multiple tumor cells (19). Thus, we speculated that NEAT1 sponges miRNAs in HFLS-RA cells. Figure 4A shows the ENCORI-predicted binding sites for miR-410-3p on NEAT1. Dual-luciferase assays revealed binding between NEAT1 and miR-410-3p in HFLS-RA cells. HFLS-RA cells transfected with NEAT1-WT and miR-410-3p mimics showed significant decrease in relative luciferase activity as compared with luciferase intensities in NEAT1-MUT-transfected HFLS-RA cells (*p* = 0.0192, Figure 4B).

Next, we investigated the effect of NEAT1 on the expression of miR-410-3p. miR-410-3p levels decreased upon overexpressing NEAT1 (*p* = 0.0196, Figures 4C,D), while downregulation reversed this phenotype in HFLS-RA cells (*p* < 0.05). This confirmed the interaction between NEAT1 and miR-410-3p, which allowed the regulation of miR-410-3p expression.

We have previously demonstrated YY1 as the direct target of miR-410-3p in FLSs from patients with RA. Thus, we determined the effect of NEAT1 on YY1 expression in HFLS-RA cells. As shown in Figures 4E–J, there was an increase in the mRNA and protein levels of YY1 upon the regulation of NEAT1 (both *p* < 0.05), while the downregulation of NEAT1 reduced the expression of YY1 (*p* < 0.001 and *p* < 0.05, respectively). Notably, YY1 levels were partially rescued upon co-transfecting HFLS-RA cells with NEAT1 and miR-410-3p mimics or siRNAs against NEAT1 and miR-410-3p inhibitor (all *p* < 0.05) as compared to those in cells transfected with NEAT1 or NEAT1 siRNAs, respectively. Taken together, these data suggest that NEAT1 positively regulates the expression of YY1 via miR-410-3p, indicating that the importance of the NEAT1/miR-410-3p/YY1 axis in the development of RA.

### NEAT1 Functions via the miR-410-3p/YY1 Axis During the Development of RA

**Figure 5:**
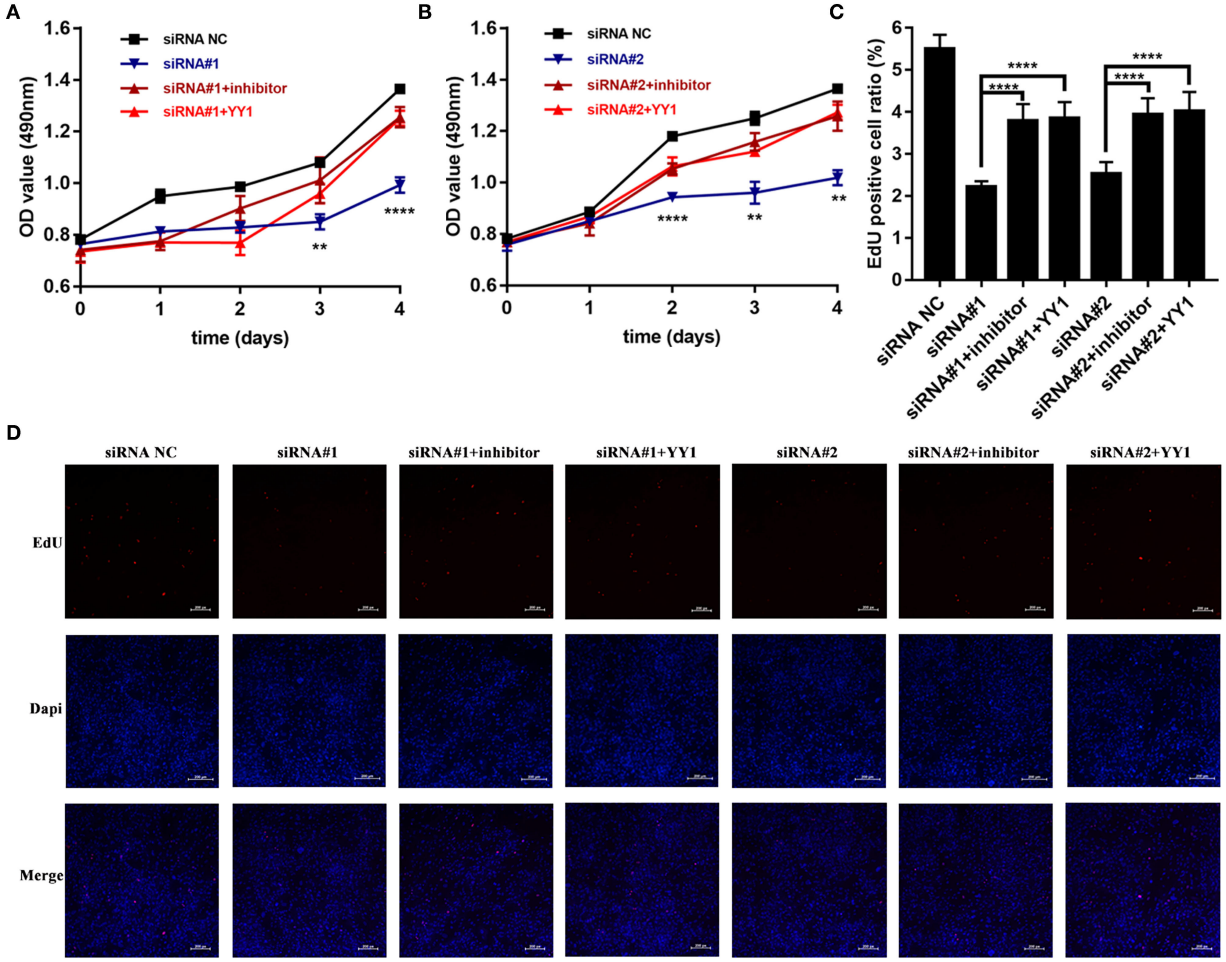
NEAT1 promotes the proliferation of HFLS-RA cells via the miR-410-3p/YY1 axis **(A,B)** The proliferation of HFLS-RA cells transfected with NEAT1 siRNAs, NEAT1 siRNAs, and miR-410-3p inhibitor, or NEAT1 siRNAs and pcDNA-YY1 for 0, 24, 48, 72, and 96 h. **(C,D)** EdU staining intensities showing the proliferation of HFLS-RA cells transfected with NEAT1 siRNAs, NEAT1 siRNAs, and miR-410-3p inhibitor, or NEAT1 siRNAs and pcDNA-YY1 for 48 h. All the experiments were independently performed in triplicates. **p < 0.01, and ****p < 0.0001, compared with the relative NC groups.

We then investigated whether NEAT1 functions in HFLS-RA cells via the miR-410-3p/YY1 axis. As shown in Figures 5A,B, siRNA-induced NEAT1 downregulation-mediated suppression of HFLS-RA cell proliferation was partially rescued by the cotransfection of NEAT1 siRNAs and miR-410-3p inhibitor (*p* < 0.01), or NEAT1 siRNAs and pcDNA-YY1 (*p* < 0.05). In accordance with this data, EdU staining showed that, compared to the cells transfected with NEAT1 siRNAs, cells cotransfected with NEAT1 siRNAs and miR-410-3p inhibitor (*p* < 0.0001, Figures 5C,D) or pcDNA-YY1 (*p* < 0.0001) exhibited an increase in the EdU-positive cells.

**Figure 6:**
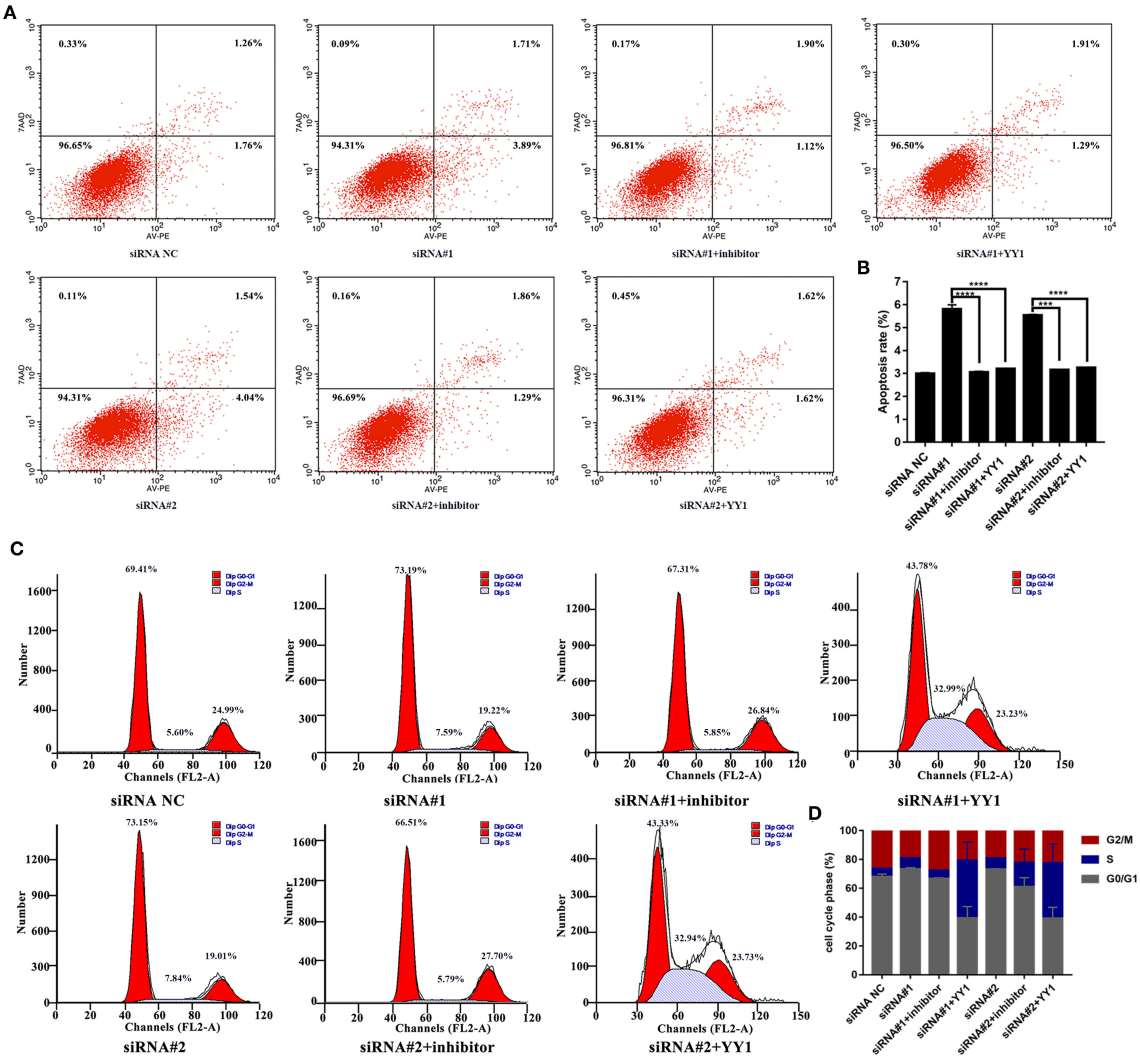
NEAT1 regulates apoptosis and cell cycle of HFLS-RA cells via the miR-410-3p/YY1 axis **(A,B)** Flow cytometric analysis showing the rates of apoptosis (%) in HFLS-RA cells transfected with NEAT1 siRNAs, NEAT1 siRNAs and miR-410-3p inhibitor, or NEAT1 siRNAs and pcDNA-YY1 for 48 h. **(C,D)** Flow cytometric analysis showing the percentage (%) of HFLS-RA cells transfected with NEAT1 siRNAs, NEAT1 siRNAs, and miR-410-3p inhibitor, or NEAT1 siRNAs and pcDNA-YY1 for 48 h in different phases. All the experiments were independently performed in triplicates. ***p < 0.001, and ****p < 0.0001, compared with the relative NC groups.

Finally, we analyzed apoptosis and cell cycle progression in HFLS-RA cells. As shown in Figures 6A,B, HFLS-RA cells cotransfected with NEAT1 siRNAs and miR-410-3p inhibitor (*p* < 0.0001) or pcDNA-YY1 (*p* < 0.001) exhibited significantly lower rates of apoptosis as compared to that in cells transfected with NEAT1 siRNAs. Furthermore, the inhibition of S-to-G2/M phase transition in HFLS-RA cells mediated by the depletion of NEAT1 was partially restored by cotransfecting NEAT1 siRNAs and miR-410-3p inhibitor (*p* < 0.001, Figures 6C,D) or pcDNA-YY1 (*p* < 0.001). Collectively, these data demonstrated that NEAT1 promotes proliferation and S-to-G2/M phase transition while suppressing apoptosis via the miR-410-3p/YY1 axis in HFLS-RA.

### Effects of NEAT1 on Other Aggressive Behaviors of HFLS-RA Cells

**Figure 7:**
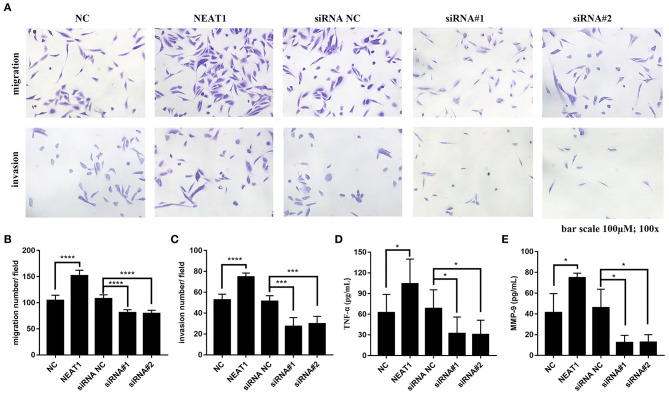
Effect of NEAT1 on migration, invasion, and inflammatory cytokines secresion of HFLS-RA **(A–C)** HFLS-RA cells were transfected with pcDNA-NEAT1 or siRNAs against NETA1, digested with trypsin, and seeded in the upper chamber of a 24-well transwell insert or Matrigel transwell insert. The cells were then incubated for 24 h for the migration and invasion assays, respectively. **(D,E)** The culture supernatants of HFLS-RA cells after transfection for 48 h were collected. The levels of TNF-α **(D)** and MMP-9 **(E)** were measured using a sandwish ELISA. All the experiments were independently performed in triplicates. *p < 0.05, ***p < 0.001, and ****p < 0.0001, compared with the relative NC groups.

In order to further support the pathogenic role for NEAT1 in RA FLSs, we performed Transwell assays to measure effects of NEAT1 on migration and invasion in RA FLSs. As shown in Figures 7A–C, upregulation of NEAT1 promoted migration and invasion of HFLS-RA cells (both *p* < 0.0001), while downregulation of NETA1 suppressed migration and invasion of HFLS-RA cells (*p* < 0.0001 and *p* < 0.001, respectively).

Subsequently, we determined inflammatory cytokines secretion from HFLS-RA cells. The data showed that upregulation of NEAT1 promoted TNF-α and MMP-9 secretion from HFLS-RAs, whereas downregulation of NETA1 suppressed TNF-α and MMP-9 secretion from HFLS-RAs (all *p* < 0.05, Figures 7D,E).

Conclusively, these data demonstrated that, besides promoting cell viability, NEAT1 promotes cell migration, invasion, and inflammatory cytokines secretion of HFLS-RA cells.

## Discussion

Patients with RA commonly manifest consistent radiographic progression in the synovial tissues. LncRNAs have recently been implicated in regulating cell proliferation, apoptosis, invasion, migration, and secretion of proinflammatory cytokines from FLSs in patients with RA (7, 20, 21). In this study, NEAT1 acted as a ceRNA for miR-410-3p that resulted in the upregulation of YY1 and exacerbated RA progression.

We first demonstrated that NEAT1 was upregulated in synovial tissues and FLSs in RA, indicating its involvement in the pathogenesis of RA; this was in accordance with the upregulation of NEAT1 in peripheral blood mononuclear cells in patients with RA (16). Previous studies have reported the oncogenic role of NEAT1 in tumorigenesis. NEAT1 overexpression promotes non-small cell lung cancer proliferation and invasion *in vitro* (22). In hepatocellular carcinoma, inhibition of NEAT1 suppresses tumor growth, migration and invasion *in vitro* (23). In high-grade serous ovarian cancer, NEAT1 promotes cell proliferation and invasion *in vitro* and enhanced tumor growth *in vivo* (24). However, the roles of NEAT1 in FLSs from patients with RA have not been clarified. Our data indicated that NEAT1 promotes cell proliferation, induces the S-to-G2/M phase transition, and suppresses apoptosis in RA FLSs *in vitro*.

LncRNAs exert their effects by mediating functional modification, histone modification and chromatin remodeling (25). Moreover, lncRNAs modulate mRNA transcription by sponging specific miRNAs like ceRNAs (26). We have previously demonstrated low expression of miR-410-3p in patients with RA; miR-410-3p exerted its protective roles by suppressing cell proliferation, reducing proinflammatory cytokines production, and inducing apoptosis in RA FLSs (17, 27). This study confirmed that NEAT1 directly bound miR-410-3p using the bioinformatics tool ENCORI and luciferase reporter assays. Furthermore, the expression of miR-410-3p was negatively correlated with the expression of NEAT1 in RA FLSs. Therefore, we hypothesized that NEAT1 functions in RA FLSs by directly regulating miR-410-3p. As expected, suppression of cell proliferation, a low proportion of G2/M phase cells, and induction of apoptosis mediated by NEAT1 knockdown was partially restored by inhibiting miR-410-3p levels, confirming that NEAT1 is an oncogene in RA FLSs and targets miR-410-3p.

As a direct target of miR-410-3p, YY1 has been confirmed to be highly upregulated in RA (17). In addition, YY1 promotes cell proliferation, migration and inflammation in RA (28–30). Since NEAT1 can bind to miR-410-3p, we determined the expression of YY1 and demonstrated that YY1 levels positively correlated with the expression of NEAT1. Notably, upregulation of YY1 induced by NEAT1 overexpression was partially restored by overexpressing miR-410-3p; downregulation of YY1 induced by NEAT1 knockdown was partially restored by inhibiting miR-410-3p, indicating that NEAT1 modulates the expression of YY1 by targeting miR-410-3p. Similarly, we evaluated the effects of YY1 on the biological functions of NEAT1 in RA. The suppressed cell proliferation and induction of apoptosis mediated by the depletion of NEAT1 was partially restored by YY1 overexpression. Unlike miR-410-3p, YY1 was involved more during the G0/G1-to-S phase transition; therefore, the proportion of G2/M phase RA FLSs was relatively high.

Besides pro-proliferative and anti-apoptotic roles, we further explored the effects of NETA1 on other aggressive phenotypes of RA FLSs. Our data demonstrated that NEAT1 promotes cell migration and invasion of RA FLSs *in vitro*. MMPs play irreplaceable roles in cell migration and invasion (31). Therefore, we further examined whether NEAT1 regulated MMPs secretion from RA FLSs. As expected, upregulation of NEAT1 promotes MMP-9 production of RA FLSs, suggesting that NEAT1 might promote cell migration and invasion via regulating MMP-9 levels. Furthermore, we found that NEAT1 promotes TNF-α secretion in RA FLSs, indicating that NEAT1 exerts pro-inflammatory roles in RA FLSs.

Our studies have some limitations. The expression of NEAT1 in synovial tissues was performed in a relatively small group of patients with RA. In addition, animal experiments validating the effects and mechanism of NEAT1 should be conducted in future study.

Overall, our results showed that NEAT1 negatively regulates miR-410-3p activity by binding to it like a ceRNA, thereby upregulating YY1. NEAT1 promotes cell proliferation, migration, invasion and inflammatory cytokines secretion, induces high S-to-G2/M phase transition, and suppresses apoptosis in RA FLSs. Thus, the NEAT1 may serve as an efficient diagnostic and therapeutic target for patients with RA.

## Data Availability Statement

All datasets presented in this study are included in the article/supplementary material.

## Ethics Statement

The studies involving human participants were reviewed and approved by the ethics committee of the Shengjing Hospital of China Medical University. The patients/participants provided their written informed consent to participate in this study.

## Author Contributions

NZ, LY, LH, XY, and NX: conception and design of the research. YW: acquisition of data and drafting the manuscript. YW and SZ: analysis and interpretation of data, NX: statistical analysis. NZ and LY: revision of manuscript for important intellectual content. All authors contributed to data analysis, drafting and revising the article, gave final approval of the version to be published, and agree to be accountable for all aspects of the work.

## Conflict of Interest

The authors declare that the research was conducted in the absence of any commercial or financial relationships that could be construed as a potential conflict of interest.
